# Multimodal creativity assessments following acute and sustained microdosing of lysergic acid diethylamide

**DOI:** 10.1007/s00213-024-06680-z

**Published:** 2024-09-05

**Authors:** Robin J. Murphy, Rachael L. Sumner, Kate Godfrey, Acima Mabidikama, Reece P. Roberts, Frederick Sundram, Suresh Muthukumaraswamy

**Affiliations:** 1 School of Pharmacy, Faculty of Medical and Health Sciences, University of Auckland, Auckland, New Zealand; 2 Centre for Psychedelic Research, Division of Psychiatry, Department of Brain Sciences, Imperial College London, London, UK; 3 School of Psychology, Faculty of Science, University of Auckland, Auckland, New Zealand; 4 Centre for Brain Research, University of Auckland, Auckland, New Zealand; 5 Department of Psychological Medicine, Faculty of Medical and Health Sciences, University of Auckland, Auckland, New Zealand

**Keywords:** Creativity, Psychedelics, Microdosing divergent thinking, Convergent thinking, Lysergic acid diethylamide

## Abstract

**Introduction:**

Enhanced creativity is often cited as an effect of microdosing (taking repeated low doses of a psychedelic drug). There have been recent efforts to validate the reported effects of microdosing, however creativity remains a difficult construct to quantify.

**Objectives:**

The current study aimed to assess microdosing’s effects on creativity using a multimodal battery of tests as part of a randomised controlled trial of microdosing lysergic acid diethylamide (LSD).

**Methods:**

Eighty healthy adult males were given 10 µg doses of LSD or placebo every third day for six weeks (14 total doses). Creativity tasks were administered at a drug-free baseline session, at a first dosing session during the acute phase of the drug’s effects, and in a drug-free final session following the six-week microdosing regimen. Creativity tasks were the Alternate Uses Test (AUT), Remote Associates Task (RAT), Consensual Assessment Technique (CAT), and an Everyday Problem-Solving Questionnaire (EPSQ).

**Results:**

No effect of drug by time was found on the AUT, RAT, CAT, or EPSQ. Baseline vocabulary skill had a significant effect on AUT and RAT scores.

**Conclusions:**

Despite participants reporting feeling more creative on dose days, objective measurement found no acute or durable effects of the microdosing protocol on creativity. Possible explanations of these null findings are that laboratory testing conditions may negatively affect ability to detect naturalistic differences in creative performance, the tests available do not capture the facets of creativity that are anecdotally affected by microdosing, or that reported enhancements of creativity are placebo effects.

**Supplementary Information:**

The online version contains supplementary material available at 10.1007/s00213-024-06680-z.

## Rationale

‘Microdosing’ refers to the practise of repeatedly ingesting very low doses of psychedelic drugs (Murphy et al. 2024b). Enhanced creativity is often mentioned as a subjective effect of microdosing protocols (Anderson et al. 2019a; Lea et al. 2019). This is consistent with the longstanding association of psychedelic use with creative experiences and inspiration (Fadiman 2011; Hofmann 1980; Sessa 2008). Despite this, there have been limited attempts to measure creativity in clinical assessments of microdosing, and none have found any significant effect on the measures used (Bershad et al. 2019; Cavanna et al. 2022; Molla et al. 2023). However, creativity testing is contentious and assessments of the kind used in these microdosing studies have been criticised elsewhere for a lack of construct validity and relevance to wider creative processes (Amabile 1996; Kaufman et al. 2008; Zeng et al. 2011). There remains a need in the field to determine whether this gap between subjective reports and objective measures of creativity in the clinic is due to placebo effects, or the complications in objectively measuring such an elusive concept as creativity. The aim of the present study is to address this gap by assessing creativity a placebo-controlled microdosing study using a multimodal battery of tests.

Indeed, in the field of creativity assessment there are conflicting arguments as to how to define and test creativity (Said-Metwaly et al. 2017). It is most commonly described as the cognitive ability to produce ideas and physical products that are both original and effective (Guilford 1967; Mednick 1962; Runco and Jaeger 2012). Within this definition, while both novelty and some degree of usefulness of the output are required, it has been argued that novelty is the primary factor, and that usefulness is secondary and only of importance when outputs are highly novel (Diedrich et al. 2015). The relative weighting of these values in practise are also context-dependant: an architect’s ideas would require a high degree of functionality, but a painter may have little interest in utility. The bipartite definition is mirrored in one of the leading theories of the cognitive processes underlying creativity: that there is a degree of unconstrained or ‘divergent’ thinking which generates novel response to a given set of conditions, accompanied by a process of constrained or ‘convergent’ thinking in which these responses are assessed for their utility (Guilford 1967). As such, the dominant forms of creativity testing broadly fall into the categories of (1) divergent thinking tasks, (2) convergent thinking tasks, (3) assessment by others, and (4) assessment by self (Kaufman et al. 2008). The existence of dissociable cognitive states involved in creative thinking have to some extent been validated by cognitive neuroscience, as performance of creative tasks have been shown to be linked to an interplay between the default mode network (DMN) and executive control network (ECN) (Beaty et al. 2015; Ellamil et al. 2012; Liu et al. 2015). The DMN is typically associated with generative thought such as mind wandering (Raichle et al. 2001), and the ECN is typically more active during goal-oriented tasks (Seeley et al. 2007). While the DMN and ECN are frequently seen to be functionally distinct, their coupling during creative tasks is theorised to be due to the dual process of idea generation and selection in divergent and convergent thinking respectively (Beaty et al. 2016).

One framework for considering creative thought is the Dynamic Frameworks of Thought (DFT) matrix (Christoff et al. 2016; Girn et al. 2020). Within this matrix, mental states can be located along axes of weak to strong deliberate and autonomic constraints. The dual processes of creative thought can be described in this matrix as shifting between periods of low and high constraints during spontaneous and goal-directed thought in idea generation and evaluation phases respectively. Within this framework, Girn et al. (2020) describe psychedelic states as spontaneous thought with low automatic and deliberate constraints, existing in a continuum with mind wandering and dreaming. Full dose psychedelic ‘trips’ tend to occasion visual hallucinations, hyper-associative thinking, enhanced cognitive flexibility, and altered meaning attribution and sense of self (Carhart-Harris et al. 2012, 2016; Doss et al. 2021; Family et al. 2016). Related to these perceptual effects, psychedelic drugs have been theorised to enhance creativity via facilitating unconstrained idea generation, while in parallel impeding the level of deliberate constraint necessary to execute effective evaluation of these ideas (Sayalı and Barrett 2023; Wießner et al. 2022). Similar frameworks for understanding psychedelics’ proposed creative effects have focused on a trade-off between cognitive stability/persistence vs. flexibility (Prochazkova and Hommel 2020; Sayalı and Barrett 2023). A commonality in these frameworks is an emphasis on unconstrained thought that is relatively free of top-down cognitive control. The following will review what is known about these processes from first the high and then low dose studies of creativity under psychedelics.

Tests of the effects of full psychedelic doses on creativity date back to the ‘first wave’ psychedelic research of the mid-20th century (for review see: Fadiman 2011; Janiger and de Rios 1989; Prochazkova and Hommel 2020; Sessa 2008). However, their low rigour by contemporary standards (lacking control groups, low sample size, lacking objective outcome measures etc.) means that results should be interpreted with caution. Preliminary creativity studies in the contemporary era of psychedelic research appear to show effects in line with the DFT’s positioning of acute psychedelic states as being conducive to generative, but not evaluative processes of creativity (Girn et al. 2020). Consistent with the idea of increased associativity, high doses of LSD has been shown to increase the spread of semantic information that is activated by a trigger stimuli (associative spread) (Family et al. 2016). Indeed, another LSD study highlights that while measures of novelty and originality (divergent thinking), and symbolic thinking are enhanced, measures of organisation and utility of ideas (convergent thinking) were decreased (Wießner et al. 2022). This has also been reported with other classic psychedelic compounds, a prospective trial of participants before and during an ayahuasca ceremony found that while one measure of divergent thinking was unchanged during the acute phase of the drug, another was enhanced, and that convergent thinking was decreased (Kuypers et al. 2016). The biological mechanism of psychedelics’ effects on creativity has not been established, although altered dopaminergic signalling has been proposed as a plausible candidate, which can account for both increased divergent and decreased convergent thinking during the acute phase (Sayalı and Barrett 2023). Notably, psychedelic states are characterised by increased between-network coupling of typically functionally distinct networks including the DMN with control networks (Daws et al. 2022; Roseman et al. 2014), similarly to the coupling observed during unmedicated creative processes (Beaty et al. 2015; Ellamil et al. 2012; Liu et al. 2015).

Persisting enhancements of creativity following acute psychedelic ‘trips’ could be of benefit to creative or problem-solving practises, however studies of the post-acute periods after full doses of psychedelics have shown conflicting results. One prospective study tracking participants before and the day and week after an ayahuasca ceremony found that convergent thinking was increased a week after the ceremony, while divergent thinking was lower both in the morning and the week after the ceremony (Kiraga et al. 2021). A similar prospective study following psilocybin dosing similarly found that convergent thinking was higher a week after the dose, but also found that divergent thinking was higher, not lower, the morning after (Mason et al. 2019). A subsequent controlled study of psilocybin full doses showed acute decreases in both divergent and convergent thinking, with decreased convergent thinking persisting one week after the dose, but divergent thinking increasing (Mason et al. 2021). The cause of these differences could be due to the substances used (ayahuasca vs. psilocybin) but may also be due to differences in mental state and testing environment (psychedelic retreat vs. laboratory), which have been shown to affect similar tests (Amabile 1996) and are both significant modifiers of psychedelic experiences – commonly referred to as ‘set and setting’ (Hartogsohn 2017). It is plausible that variation in acute psychedelic experiences have consequences on subsequent post-acute creative effects, as has been suggested by correlation analysis of acute functional connectivity (measurable by fMRI) to post-acute creativity performance (Mason et al. 2021). Post-acute increases in divergent thinking were predicted by disruption of within-network functional connectivity in the acute phase, and post-acute decreases in convergent thinking were predicted by increased acute connectivity between the DMN and fronto-parietal control network (FPN) (Mason et al. 2021). This provides an objective link between acute brain activity and post-acute effects on divergent and convergent thinking, highlighting the characteristic psychedelic induced network disintegration as a potential mechanism.

If we consider these findings alongside the bipartite definition of creativity, needing both novelty and utility – acute increases to divergent thinking that appear to occur during full doses of psychedelics may be of limited use in the practical enhancement of creative ability if not accompanied by the ability to accurately evaluate the effectiveness of ideas. One might consider the heightened creative process to be complete upon the cessation of psychoactive effects, where a return to normal consciousness allows for critical appraisal of the divergent thinking elicited during the acute period. However, as discussed above, post-acute results are variable and repeatedly undergoing high doses to access acute benefits that may exist would be unsustainable for most people. As such, microdosing could present an alternative to elicit some of the associative enhancements of psychedelic drugs while maintaining enough cognitive control to evaluate the utility of ideas effectively (Prochazkova and Hommel 2020). Community microdosers frequently report improved creativity in surveys (Anderson et al. 2019a; Lea et al. 2020), and in publicly posted reports of their experiences (Andersson and Kjellgren 2019; Lea et al. 2019). A retrospective assessment that compared community microdosers to non-microdosers demonstrated that microdosers had significantly higher scores on a divergent thinking task (Anderson et al. 2019b), however this is limited by potential trait confounds between populations. One open label field study of microdosing psilocybin truffles found post-acute enhancements to both divergent and convergent thinking (Prochazkova et al. 2018). One prospective study of community microdosers over six weeks found no change to a subjective scale of creative personality but saw a significant increase in ratings of feeling creative on dose days relative to non-dose days (Polito and Liknaitzky 2022). While this evidence is all from uncontrolled studies, an increase in dose day self-rated feelings of creativity was replicated in our placebo-controlled study of LSD microdosing (MDLSD trial) reported in Murphy et al. (2023). We also found dose day increases in ratings of energy, connectedness, happiness, and wellness, which does present the possibility that increased ratings of feeling creative could be part of a general uplift in positive mood. The present study reports data also collected during this trial.

Despite the volume of self-reported and prospective evidence for microdosing’s effects on creativity, both divergent and convergent thinking tasks during and after laboratory-controlled microdoses have never shown a significant effect (Bershad et al. 2019; Cavanna et al. 2022; Molla et al. 2023). The tasks used to date are standard measures of divergent and convergent thinking, including the Alternate Uses Test (AUT) (Guilford 1967) and the Remote Associates Task (RAT) (Mednick 1968). The AUT gives participants the names of various household objects, and asks them to think of as many uncommon uses for each item as they can and is considered a measure of divergent thinking (Guilford 1967). The RAT gives participants three words which are linked by an undisclosed common concept, and participants are asked to think of this linking word in order to measure convergent thinking and associative spread (Bowden and Jung-Beeman 2003; Mednick 1968). It is possible that these tasks used in the controlled studies to-date are not entirely adequate for capturing the experiences of creativity reported by microdoses. Creativity tasks of this kind have been criticised for giving an incomplete picture of creative processes by being too domain specific, presenting closed rather than open problems, and failing to adequately capture the criteria of efficiency and the phenomenon of insight (Amabile 1996; Kaufman et al. 2008; Said-Metwaly et al. 2017; Wakefield 1987; Zeng et al. 2011).

An alternative approach that may have more construct validity in the microdosing context is testing creative output, such as art or creative writing using the Consensual Assessment Technique (CAT) (Amabile 1996). In this task, participants are given a practical art or writing assignment, and outputs are then scored for their level of creativity by a panel of experts. These experts are asked to rate both the creative and technical elements of the piece in relation to the overall performance of the group. This approach is argued to be more valid than tests like the RAT and AUT because it is a measure of applied creative activities which accounts for effectiveness by being externally rated, and should therefore be considered the gold standard of creativity testing (Amabile 1996; Kaufman et al. 2008). The limitations of this approach however are that the tests are still domain-specific, and they may be affected by participants’ own proficiency in that particular domain (Zeng et al. 2011). The CAT task has not previously been administered during any studies of microdosing psychedelics. The degree to which creativity is domain-specific or domain-general has been debated, but it is reasonable to conclude that creative processes require elements of both types of abilities and that creative testing in only one domain is inadequate to get a complete picture (Amabile 1996; Lubart and Guignard 2004; Plucker and Beghetto 2004).

In order to overcome domain specificity in creativity testing, the current study implemented a multimodal approach to test across several domains and cognitive processes, an approach that has been recommended in the literature (Kaufman et al. 2008; Lubart and Guignard 2004). Our creativity battery included the RAT and AUT in order to replicate existing studies of microdosed psychedelics (Bershad et al. 2019; Cavanna et al. 2022; Molla et al. 2023), a visual art CAT task to add a different modality with more practical creative output and a degree of assessment by others, and daily VAS ratings of participants self-rated feelings of being creative (reported previously in Murphy et al. (2023). In this way, the MDLSD study covered all the four assessment domains identified by Kaufman et al. (2008) – divergent thinking, convergent thinking, assessment by others, and assessment by self. Despite this, our multimodal battery still did contain an ecological gap in that the domain-specific tasks present relatively closed problems with little relation to how participants likely experience creativity in their daily lives, and the creativity VAS rating gave little depth as to these experiences. To address this gap, a bespoke Everyday Problem-Solving Questionnaire (EPSQ) was developed in-house which asked participants about instances that required novel problem solving in their everyday lives and asked them to rate both their ability to generate a solution, as well as the utility of that solution, to capture both unconstrained idea generation, and constrained idea evaluation.

The aim of the following study was to robustly test for acute and durable effects of microdosing on creativity in the MDLSD trial using a visual art CAT, linguistic RAT and AUT tasks, as well as the bespoke EPSQ. The daily VAS ratings of creativity have already been presented in Murphy et al. (2023) but will be revisited in the discussion. Given the paucity of robust existing evidence, we did not have specific hypotheses.

## Methods

The MDLSD trial consisted of eighty healthy male participants randomised into either LSD (*n* = 40) or placebo (*n* = 40) groups. Full inclusion and exclusion data is included in the Supplementary Materials. Creativity tasks took approximately 30–45 min and were first undertaken at a drug-free Baseline session (Table 1). The order of tasks was AUT, CAT, RAT. Task order was not counterbalanced within the battery. Approximately one week later, participants returned for a Treatment session, in which they were administered their first 10 µg LSD microdose under supervision and creativity tasks were repeated at 240 min after taking the dose. Peak subjective effects of 10 µg LSD have previously been observed at 150 min post-dose (Holze et al. 2020), however as the creativity tasks were secondary measures, priority scheduling at peak of effects was given to primary EEG measures (Murphy et al. 2021, 2024a). Pharmacodynamic data from the present study (currently in review) show that at 240 min participants’ ratings of feeling an effect were at 81.7% of the maximum observed effect, indicating that acute effects could still be regarded as being present at this time point (Morse et al. 2024). Participants then self-administered 13 subsequent doses at home on an every-third-day protocol with some flexibility. Two days after their final dose, participants returned for a drug-free Final visit and creativity tasks were repeated. Participants were run in four waves of 19-21 between 2021 and 2022.

**Table 1:**
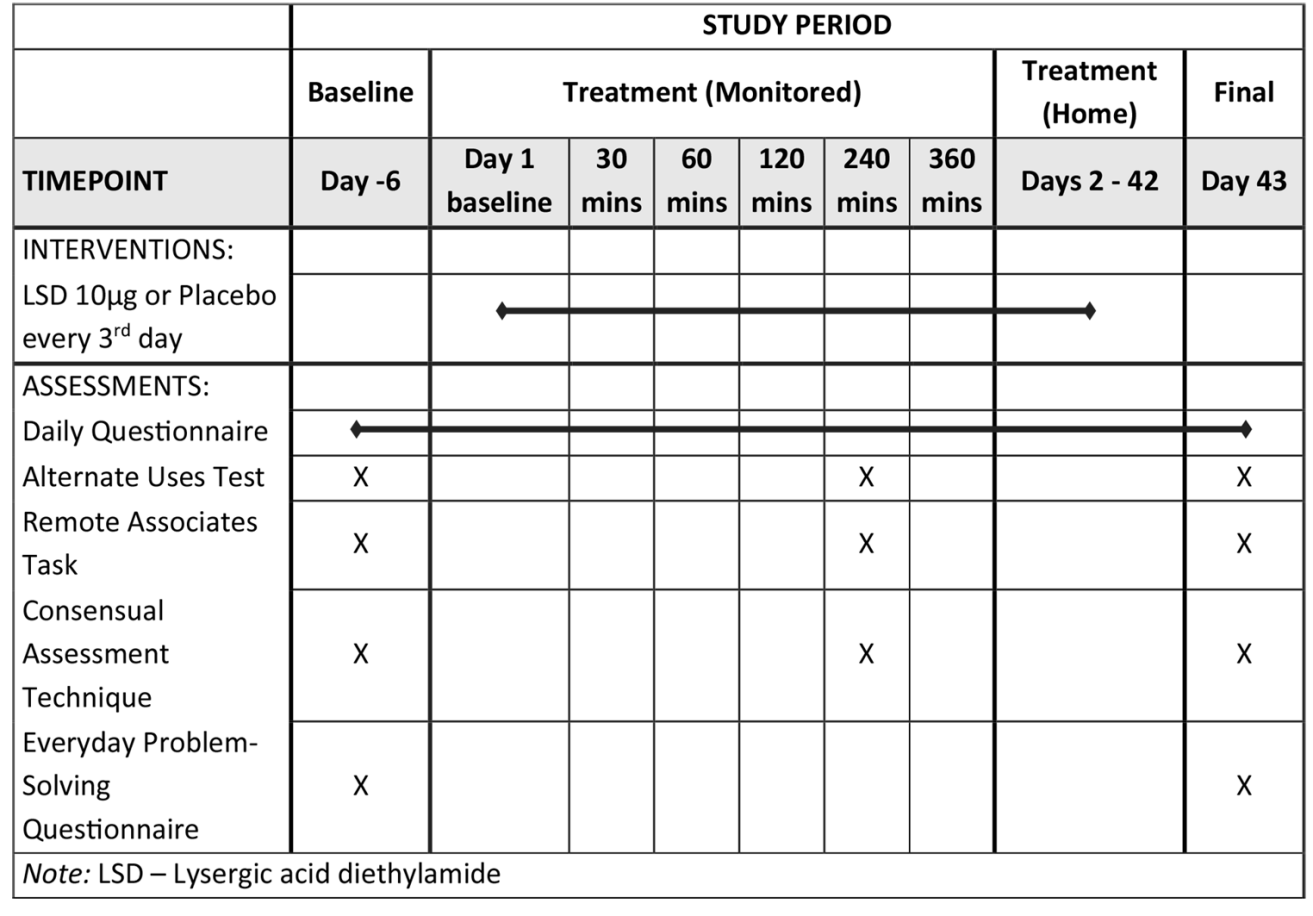
Schedule of assessments

### Alternate Uses Test

AUT and RAT tasks were initially administered via Qualtrics XM (https://www.qualtrics.com) on an iPad in a closed room at the Baseline, Treatment (240 min after dosing) and Final visits. However during the second wave of the trial (out of four), local Covid-19 restrictions meant that as much of the data needed to be collected remotely as possible, so some AUT and RAT tasks were completed remotely by participants on the correct dates but in their home environment with their own devices to access Qualtrics (see Murphy et al. (2023) for full description of Covid-19 related adaptations of the protocol). The tasks continued to be administered on-site for all Treatment visits.

Three versions of the AUT task were administered in counterbalanced order between participants. Each version contained three different everyday words. Participants had two minutes to think of as many different uses for each word as they could. Words were presented in a randomised order. Full instructions and the items used are given in the Supplementary Materials.

At the completion of the trial, the answers for each AUT item were then coded for the following outcome measures:

1. Frequency: the total number of responses given.
2. Flexibility: the number of different categories of responses given.
3. Elaboration: the degree to which the participant elaborates on their response (one point for each additional detail).
4. Originality: the uniqueness of the response (one point for responses given by less than 15% of the sample, two points if given by less than 10%, three points if given by less than 5%).

Two independent raters coded each item for the first 10 participants, after which Cronbach’s alpha was calculated using the *alpha* function from the *psych* package in R. If an alpha value greater than 0.7 was achieved, only one rater then continued with rating the entire dataset.

### Remote Associates Task

Three testing versions of the RAT were administered in counterbalanced order, with one practice version being pre-administered to all participants at the Screening visit. Each version consisted of 20 trials of three words each, with the correct answer being a fourth word that conceptually links to each of the others. In the practice version, participants were shown the correct answer after each trial, but they were not shown the answer in the testing versions. Trials were drawn from those used by Bershad et al. (2019), and adapted to the New Zealand context by replacing inappropriate trials with items of similar difficulty from Bowden and Jung-Beeman (2003). Instructions for the task are given in the Supplementary Materials.

Each item is designed with one correct answer expected, however answers were also reviewed for reasonable but unexpected answers (accepted with agreement between two raters). Outcome measures were the number of correct/reasonable responses and the number of trials attempted.

### Consensual Assessment Technique

CAT tasks were administered in a closed room at the Baseline, Treatment (~ 240 min after dosing) and Final visits. Per the instructions of Amabile (1996), participants were given 15 min to create ‘a design that conveys a sense of silliness’. Materials for making the designs were six coloured pieces of construction paper (red, blue, green, yellow, orange, and white) in a brown paper envelope, a glue stick, and scissors. Designs were photographed by the study team. When aspects of the design were not able to be captured by photography (e.g. there was some kinetic or conceptual component) the study team recorded a brief description. At the completion of the trial, 16 secondary school art teachers were recruited to rate the designs using Qualtrics XM (https://www.qualtrics.com). To prepare them for the task, raters were first shown a representative sample of 10 randomly selected designs and instructed to imagine how they would order them from lowest to highest in terms of ‘creativity’ and ‘technical goodness’. Images of each design in the total dataset were then presented in random order with instructions to rate each design on creativity and technical goodness on a continuous VAS scale from 0 to 10 with 0 being labelled as ‘One of the lowest’, 5 labelled as ‘Average’, and 10 labelled as ‘One of the highest’. If an additional description had been provided by the study team it was given underneath the image. Raters were given a brief description of the task and told to rate designs in relation to the group overall, rather than according to their general standards of artistic quality. They were also instructed to consider creativity and technical goodness as independent categories.

### Everyday problem-solving questionnaire

A bespoke questionnaire was developed to more thoroughly assess subjective experiences of creativity during the trial, beyond the ‘feeling creative’ VAS ratings reported in Murphy et al. (2023). As part of the battery of psychometric assessments at the Baseline visit, participants were asked to nominate ‘a situation in your job, hobby, or everyday life when you encounter problems that require you to think of novel (new) solutions’. Participants were then asked to consider how difficult it was to think of solutions to those problems (from ‘extremely easy’ to ‘extremely difficult’) when they had encountered that problem in the last month, and how difficult it was to visualise solutions to those problems (from ‘extremely easy’ to ‘extremely difficult’) – the mean of which was an ‘idea generation difficulty’ scale. They were also asked how practical the solutions that they came up with were (from ‘not at all practical’ to ‘extremely practical’), and how satisfied they were with the solutions that they came to (from ‘not at all satisfied to ‘extremely satisfied) – the mean of which was an ‘idea evaluation’ scale. Participants rated their answers to these four questions on 100-point scales from − 50 to + 50. At the Final measure point, participants were reminded of the situation that they had nominated in the Baseline session and again asked the same four questions regarding their experience of problem solving in the past month. Outcome measures were idea generation and idea evaluation scores.

### Statistical analysis

Each task and questionnaire was analysed with a linear mixed effects model using the *lmerTest* package in R (Kuznetsova et al. 2017) with Group and Visit treated as fixed effects, and Participants as random effect. Linear mixed effects modelling was chosen due to the ability to accommodate missing data without excluding participants, and to account for the random effects of participants variable abilities at the tasks. Language ability was controlled for in the AUT and RAT analyses by including scores on the NIH Toolbox Picture Vocabulary Test (Weintraub et al. 2013) as a fixed effect covariate. The vocabulary test was administered via the NIH Toolbox iPad app at the Baseline visit (National Institutes of Health 2019). Significant results were uncorrected and considered exploratory. Post-hoc analyses were conducted by calculating the estimated marginal means using the *emmeans* package in R. To check whether the change in administration location (home versus in the lab) was significant, a follow up analysis was conducted on the RAT and AUT scores which also included study wave (1–4, see Supplementary Materials for description of varying study wave conditions), as a fixed effect. All analyses were based on intention-to-treat. Effect sizes were calculated as partial Eta squared (proportion of variance explained by fixed effects and interactions separately; η_p_^2^) using the *effectsize* package in R.

The inter-rater reliability of AUT and CAT ratings was tested by computing Cronbach’s alpha using the *alpha* function from the *psych* package in R. Separate alpha values were computed for ‘creativity’ and ‘technical goodness’ ratings in the CAT. In the AUT, alpha was calculated for flexibility and originality given that these two ratings are subjective. An alpha value over 0.7 was considered acceptable.

## Results

### Participants

Analyses were intention-to-treat and included all completed tasks. Of the 80 enrolled participants, 75 completed the full course of doses (placebo = 39, LSD = 36), however withdrawn participants completed all tasks and questionnaires and were included in analysis, with the exception of one CAT task in the placebo group at the Final visit. Full details of withdrawals are given in Murphy et al. (2023). Additionally, there were three instances of missing/corrupted data in the AUT during the Treatment session in the placebo group, and in the LSD group there was one instance of missing/corrupted data RAT task at each of the Baseline and Final sessions. Table 2 gives the size of each dataset for each session by group.

**Table 2:** Dataset size for each group and session for creativity measures

	Placebo				LSD		
Measure	**Baseline**, ***n*(%)**	**Treatment**, ***n*(%)**	**Final**, ***n*(%)**		**Baseline**, ***n*(%)**	**Treatment**, ***n*(%)**	**Final**, ***n*(%)**
AUT	40 (100)	37 (92.5)	40 (100)		40 (100)	40 (100)	40 (100)
RAT	40 (100)	40 (100)	40 (100)		39 (97.5)	40 (100)	39 (97.5)
CAT	40 (100)	40 (100)	39 (97.5)		40 (100)	40 (100)	40 (100)
EPSQ	40 (100)	40 (100)	40 (100)		40 (100)	40 (100)	40 (100)

*Note* AUT – Alternate Uses Test; CAT – Consensual Assessment Technique; EPSQ – Everyday Problem-Solving Questionnaire; RAT – Remote Associates Task

### Alternate Uses Test

**Fig. 1:**
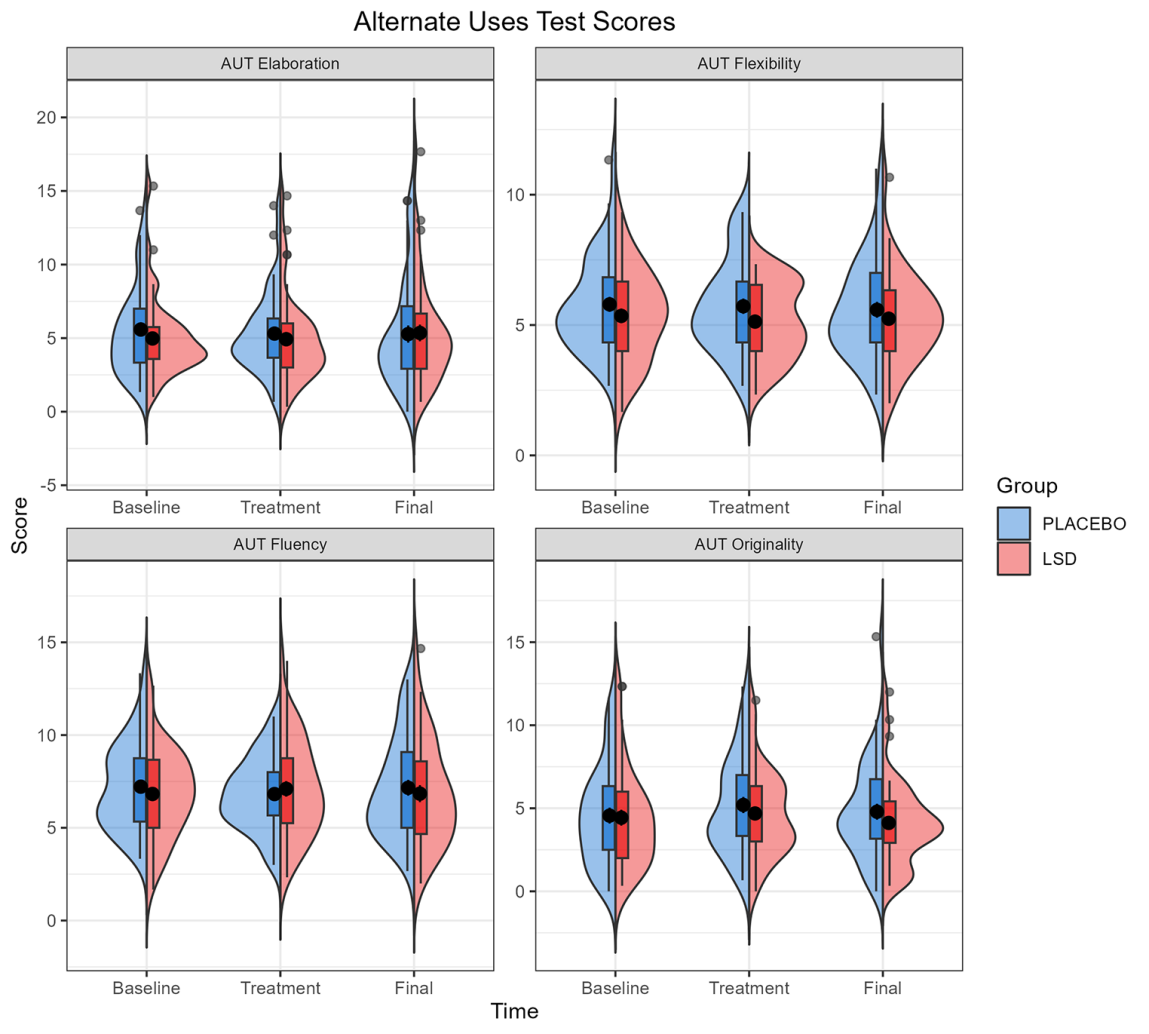
Alternate Uses Test (AUT) scores for elaboration, flexibility, fluency, and originality by Group at Baseline, Treatment, and Final visits. Box plots show the inter quartile range (IQR), with values over 1.5 IQR from the hinge represented as outlier points

Analysis of the AUT in a Group (Placebo vs. LSD) x Visit (Baseline vs. Treatment vs. Final) linear mixed effects model with Vocabulary as a fixed effect showed no interaction effect of Group x Visit on fluency, flexibility, elaboration, nor originality. There was no main effect of Group or Visit. There was a significant effect of Vocabulary on fluency (*F* = 5.99, *p* = 0.017, *η*_*p*_^*2*^ = 0.072), flexibility (*F* = 20.42, *p* < 0.001, *η*_*p*_^*2*^ = 0.207), elaboration (*F* = 9.78, *p* = 0.002, *η*_*p*_^*2*^ = 0.111) and originality (*F* = 11.63, *p* = 0.001, *η*_*p*_^*2*^ = 0.129) with the largest effect being on flexibility (the number of different categories of responses given). Post-hoc analysis of the effect of Vocabulary showed that the difference in estimated marginal means between the 25th and 75th percentiles was − 0.72 for fluency (SE = 0.29, *p* = 0.017), -0.83 for flexibility (SE = 0.18, *p* < 0.001), -1.09 for elaboration (SE = 0.35, *p* = 0.003), and − 0.92 for originality (SE = 0.29, *p* = 0.001). In all cases this indicates that AUT scores were higher for participants with greater vocabulary scores. Table 3 gives the test statistics for the main and interaction effects for fluency/flexibility/elaboration/originality and Fig. 1 gives the mean scores across each visit. Follow up analysis did not find an effect of block in any of the AUT measures.

**Table 3:** Main and interaction effects in the AUT analysis of Group x Visit + Vocabulary

	Main effect Group				Main effect Visit				Main effect Vocabulary				Interaction effect Group x Visit			
AUT Measure	F	*p*	η_*p*_^2^		F	*p*	η_*p*_^2^		F	*p*	η_*p*_^2^		F	*p*		η_*p*_^2^
Fluency	0.12	0.730	0.002		0.02	0.981	< 0.001		5.99	0.017	0.072		1.10	0.336		0.014
Flexibility	2.60	0.111	0.033		0.45	0.637	0.006		20.42	< 0.001	0.207		0.33	0.716		0.004
Elaboration	0.33	0.567	0.004		0.20	0.822	0.003		9.78	0.002	0.111		0.57	0.569		0.007
Originality	0.97	0.329	0.012		1.13	0.326	0.014		11.63	0.001	0.129		0.31	0.731		0.004

*Note: *AUT – Alternate Uses Test. ηp^2^ – Partial Eta squared

### Remote Associates Task

**Fig. 2:**
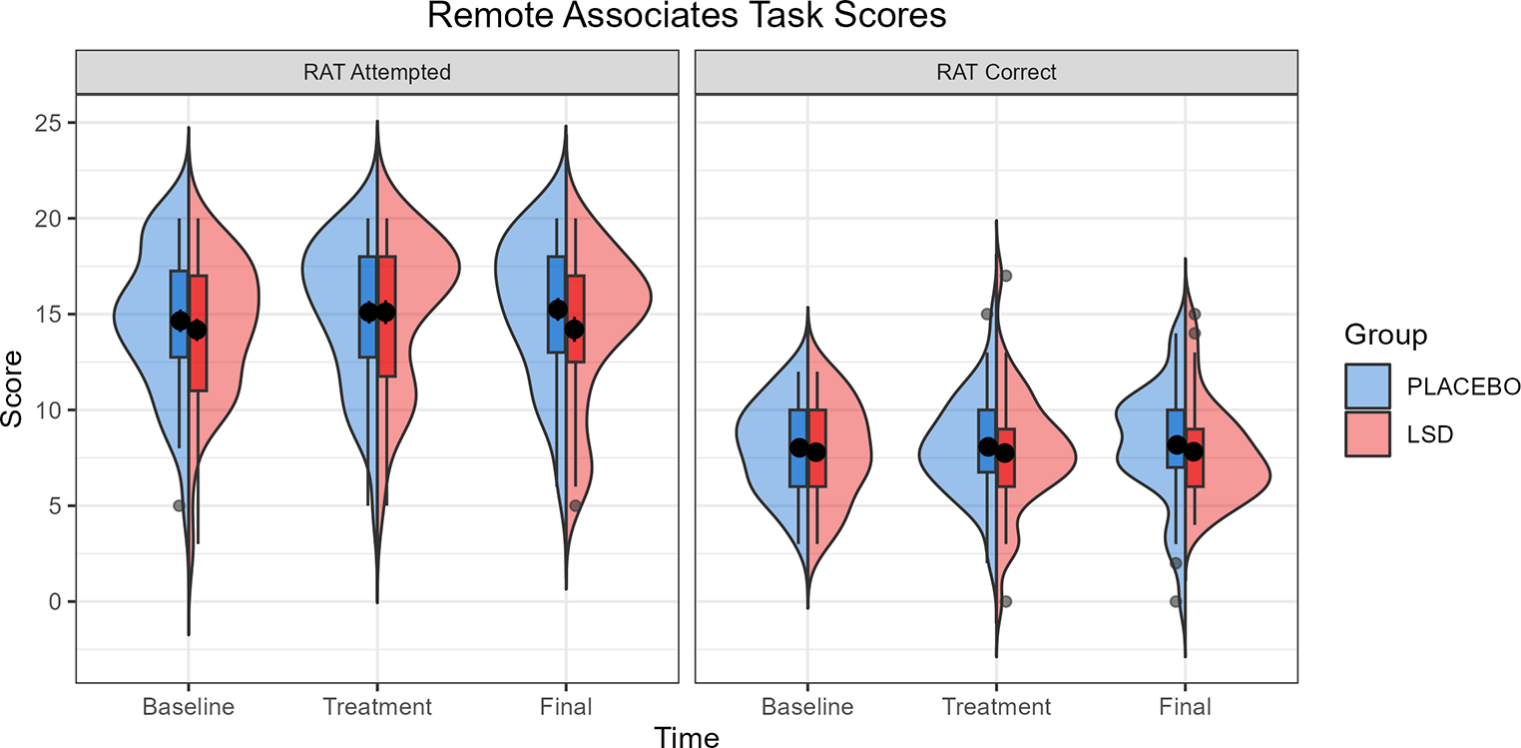
Remote Associates Taks (RAT) scores for number attempted and number correct by Group at Baseline, Treatment, and Final visits. Box plots show the inter quartile range (IQR), with values over 1.5 IQR from the hinge represented as outlier points

Analysis of the RAT test in a Group (Placebo vs. LSD) x Visit (Baseline vs. Treatment vs. Final) linear mixed effects model with Vocabulary as a fixed effect showed no interaction effect of Group x Visit on either the number of items correct or attempted. There was no main effect of Group or Visit. There was a significant effect of Vocabulary on the number of items correct (*F* = 45.04, *p* < 0.001, *η*_*p*_^*2*^ = 0.355), but not the number of items attempted. Post-hoc analysis of the effect of Vocabulary on the number of items attempted showed that the difference in estimated marginal means between the 25th and 75th percentiles was − 1.47 (SE = 0.22, *p* < 0.001) indicating that the number of RAT items correct was greater for participants who had a higher baseline vocabulary. Follow up analysis did not find an effect of block in any of the measures. Table 4 gives the test statistics for the main and interaction effects for the number correct/attempted and Fig. 2 gives the mean scores across each visit.

**Table 4:** Main and interaction effects in the RAT analysis of Group x Visit + Vocabulary

	Main effect Group				Main effect Visit				Main effect Vocabulary				Interaction effect Group x Visit			
RAT Measure	F	*p*	η_*p*_^2^		F	*p*	η_*p*_^2^		F	*p*	η_*p*_^2^		F	*p*		η_*p*_^2^
Number correct	1.06	0.306	0.013		0	0.995	< 0.001		45.04	< 0.001	0.355		0.05	0.949		0.001
Number attempted	0.44	0.507	0.006		1.59	0.208	0.020		0.58	0.448	0.007		1.05	0.354		0.013

*Note: *RAT – Remote Associates Task.η_p_^2^ – Partial Eta squared

### Consensual Assessment Technique

**Fig. 3:**
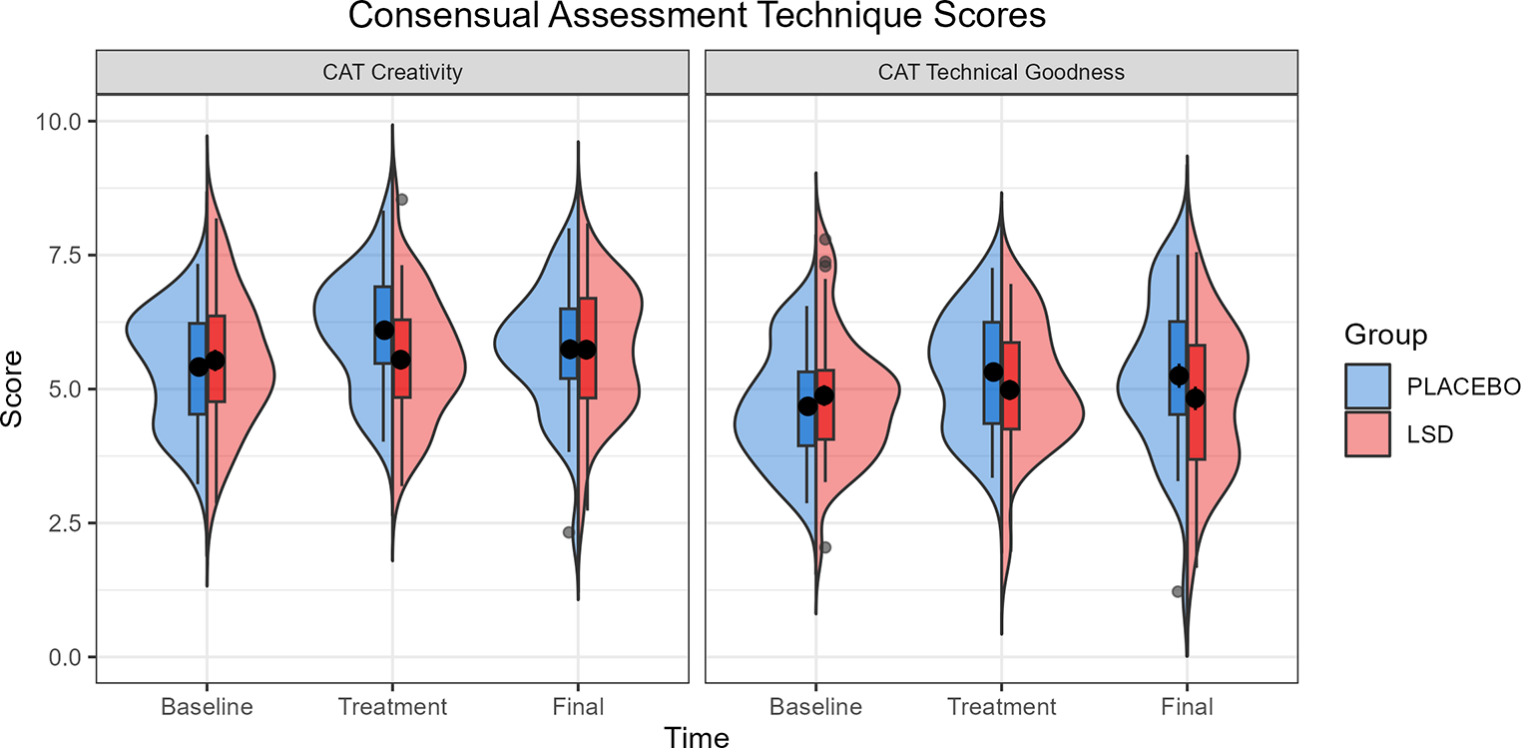
Consensual Assessment Technique (CAT) scores for creativity and technical goodness by Group at Baseline, Treatment, and Final visits. Box plots show the inter quartile range (IQR), with values over 1.5 IQR from the hinge represented as outlier points

CAT raters were 16 secondary school art teachers (11 female, five male) with mean teaching experience of 8.4 years and mean years as an artist of 15.6 years. Analysis of the CAT in a Group (Placebo vs. LSD) x Visit (Baseline vs. Treatment vs. Final) linear mixed effects model showed no interaction effect of Group x Visit on creativity nor technical goodness, nor any main effects. Table 5 gives the test statistics for the main and interaction effects for the number creativity and technical goodness and Fig. 3 gives the mean scores across each visit.

**Table 5:** Main and interaction effects in the CAT analysis of Group x Visit

			Main effect Group				Main effect Visit				Interaction effect Group x Visit		
CAT Measure			F	*p*	η_*p*_^2^		F	*p*	η_*p*_^2^		F	*p*	η_*p*_^2^
Creativity			0.63	0.429	0.008		2.86	0.060	0.036		2.67	0.073	0.033
Technical goodness			0.87	0.354	0.011		2.87	0.060	0.036		2.27	0.107	0.028

*Note: *CAT – Consensual Assessment Technique.η_p_^2^ – Partial Eta squared

### Everyday problem-solving questionnaire

**Fig. 4:**
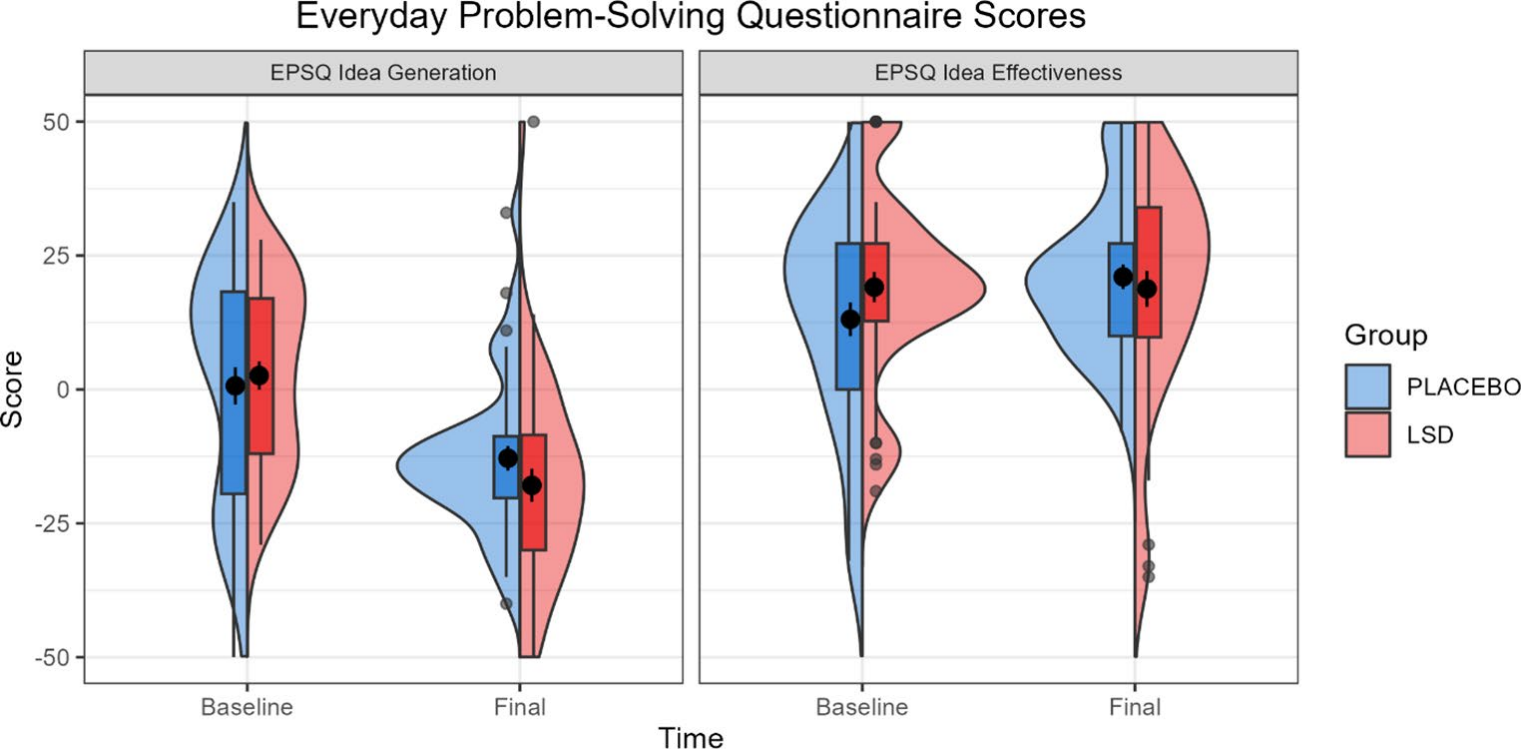
Everyday Problem Solving (EPSQ) ratings of idea generation difficulty and idea effectiveness by Group at Baseline and Final visits. Box plots show the inter quartile range (IQR), with values over 1.5 IQR from the hinge represented as outlier points

Analysis of the EPSQ test in a Group (Placebo vs. LSD) x Visit (Baseline vs. Treatment vs. Final) linear mixed effects model showed no interaction effect of Group x Visit on idea generation difficulty or idea effectiveness. There was a significant main effect of Visit on idea generation difficulty (*F* = 47.35, *p* < 0.001, *η*_*p*_^*2*^ = 0.378), but not idea effectiveness. Post-hoc analysis of the effect of Visit on idea generation difficulty showed a significant difference between Baseline and Final visit scores for both the placebo and LSD groups with a difference in estimated marginal means of 17 (SE = 2.47, *p* < 0.001) indicating that, regardless of group, participants rated idea generation as more difficult at the Baseline than at the Final visit. Table 6 gives the test statistics for the main and interaction effects for idea generation/effectiveness and Fig. 4 gives the mean scores across each visit.

**Table 6:** Main and interaction effects in the EPSQ analysis of Group x Visit

	Main effect Group				Main effect Visit				Interaction effect Group x Visit		
CAT Measure	F	*p*	η_*p*_^2^		F	*p*	η_*p*_^2^		F	*p*	η_*p*_^2^
Idea generation difficulty	0.23	0.636	0.003		47.35	< 0.001	0.378		1.99	0.162	0.025
Idea effectiveness	0.36	0.553	0.005		2.03	0.158	0.025		2.36	0.128	0.029

*Note: *EPSQ – Everyday Problem-Solving Questionnaire.η_p_^2^ – Partial Eta squared

## Discussion

This study tested the acute and durable effect of microdosing on creativity using four different modalities. Consistent with previous controlled and semi-controlled acute experiments, no interaction effect of drug group by visit was seen on divergent thinking as measured by the AUT, nor convergent thinking as measured by the RAT (Bershad et al. 2019; Cavanna et al. 2022; Molla et al. 2023). No effect was seen on externally assessed practical creativity as measured by the CAT, nor on self-assessment as measured by the EPSQ. A significant effect of vocabulary was seen on accuracy in the RAT task and on all scales of the AUT, highlighting the necessary of vocabulary as a control variable in linguistic creativity tasks. There was a significant negative effect of visit on idea generation difficulty, regardless of group, suggesting that placebo effects or an effect of being in the trial affected participant self-ratings of creativity.

The null results in this study are in contrast to the significant increase in self-rated creativity on the dose days reported in Murphy et al. (2023) and to the self-rated experiences of microdosers in the community (Anderson et al. 2019a; Andersson and Kjellgren 2019; Lea et al. 2019, 2020). The gap between the findings inside and outside the lab may simply be explained by lack of power in the laboratory studies, however there are three other possible explanations: (1) testing conditions in the lab may dampen or mask any potential creativity benefits of microdosing; (2) the tests used are not valid for testing the types of creativity enhanced by microdosing; (3) enhancements to creativity reported in the grey literature are placebo effects.

### Set and setting in creativity testing

All the tests of creativity during laboratory-supplied microdoses conducted previously have been administered in controlled environments, and it is possible that these testing conditions are not conducive to fostering creativity. Indeed environmental conditions are known to affect creativity testing outcomes (for review see: Amabile 1996; Said-Metwaly et al. 2017). Full psychedelic experiences are also greatly affected by mindset and environment – termed ‘set and setting’ in the literature (Hartogsohn 2017). It is worth nothing that in the VAS ratings of dose day creativity, the effect size for that first dosing day in the lab is negative, in contrast to the subsequent 13 home dosing days (Murphy et al. 2023). Further to this, the one microdosing study which did show increased divergent and convergent thinking was conducted following unblinded microdoses during a psychedelic event (Prochazkova et al. 2018). This may have been more conducive to fostering creativity, but also could have influenced expectations of participants and led to extra effort during the acute administration of the tasks – which will be discussed below. It could be argued that the sterile and potentially stressful clinic/laboratory environments where controlled studies such as ours and others (Bershad et al. 2019; Molla et al. 2023) took place negatively affected testing performance. One limitation of our study is that fluctuating Covid-19 restrictions meant that in some study waves the long-term RAT and AUT measures had to be administered in participants’ home environments rather than the lab, although this presented the advantage of being able to test if these conditions altered outcomes. Our follow-up analyses showed no effect of study wave on the RAT and AUT scores which suggests that study environment does not explain the lack of post-acute cumulative effects of microdosing on creativity. However, we were never able to administer creativity tasks in participants’ home environments during the acute phase of the drug, and it may still be the case that lab environments are not the appropriate setting to facilitate an effect of acute microdosing on creativity. Future parameter-finding studies could compare creativity tasks under controlled microdoses in laboratory and naturalistic environments to investigate this factor further.

### Appropriateness of tests

Creativity testing itself is a contentious issue, in part due to differences in defining creativity, and in the debated validity of the available tests to measure these constructs (Said-Metwaly et al. 2017). Tasks such as the RAT and AUT have been criticised for purporting to measure general creativity with domain-specific tasks (in this case linguistic; Amabile 1996). Their construct validity has also been challenged on the grounds that they present closed rather than open problems, do not produce creative outcomes, nor account for the high-level creative breakthroughs that are characterised by insight (Amabile 1996; Kaufman et al. 2008; Said-Metwaly et al. 2017; Wakefield 1987; Zeng et al. 2011). Divergent thinking tasks such as the AUT have also been criticised for not incorporating the critical element of effectiveness or appropriateness (Zeng et al. 2011). To mitigate this, a battery which tested across the breadth of available measures was employed in this study, as well as including one bespoke self-rated measure. None of these measures showed an effect of drug, however there are still several unanswered questions in terms of how these measures may or may not be able to capture the purported effects of microdosing on creativity.

One issue is the debate around domain-specificity versus domain-generality (Amabile 1996; Said-Metwaly et al. 2017). It has been argued that creative ability at any one task incorporates both domain-general creative abilities, as well as domain-specific proficiencies and that as such, performance in a domain-specific task may not be reflective of general abilities (Amabile 1996). Our battery of measures included linguistic and visual tasks in an attempt to get a broad picture of creative abilities across different domains, however sampled from a general population that likely had a wide variance of aptitude and experience in each of those areas. In the case of the linguistic tasks, we were able to control for this by including baseline vocabulary as a variable, however a limitation of this study is that we did not have a comparable baseline control variable for visual art ability. In the linguistic tasks, vocabulary did affect the number of correct answers in the convergent thinking task (but not the number attempted) and in all measures of the divergent thinking task. Future research using these tasks should therefore aim to have skill or experience-based control variables for each domain. It would also be interesting to repeat these measures with participants who are experts in these domains, for example artists and writers, to see if microdosing is has different effects on creativity in a task at which participants are already demonstrably skilled. Domain-specific problem-solving in participants’ area of expertise was investigated in early full dose studies (for review see: Fadiman 2011; Janiger and de Rios 1989; Prochazkova and Hommel 2020; Sessa 2008), however this has not been tested following microdoses.

Another potential issue, which affects cognitive measures more generally, is that of ‘task-impurity’ – in which tasks may test for overlapping processes which could be affected by variables such as microdosing drugs in contradictory ways (Sayalı and Barrett 2023). For example the RAT, which is generally considered to be a test of convergent thinking, is also affected by the degree of associative spread that stimuli cause in order to effectively connect disparate concepts with a common link (Mednick 1962). Associativity in the form of semantic activation has been shown to be enhanced under full doses of psychedelics (Family et al. 2016), while convergent thinking is inhibited (Kuypers et al. 2016; Mason et al. 2021; Wießner et al. 2022), therefore it is possible that performance at the RAT under psychedelic doses (potentially including microdoses) is affected by contradictory enhancements and impairments in its underlying processes.

It could be that the previously reported increase in subjective ratings of feeling creative seen in microdosers on dose days (Murphy et al. 2023) reflects a true increase in creativity, but one which is not measurable with laboratory tasks, due to the problems discussed above. However, the inverse may also be true – reports of feeling creative may simply be a feeling with no bearing on actual creative output. Elsewhere, subjective feelings of creativity have been linked to positive mood (Han et al. 2019; Zhang et al. 2020). A dose day change in happiness and wellbeing was indeed seen alongside increases in creativity in our previously-reported data (Murphy et al. 2023), therefore it is plausible that the increase in feeling creative may be more related to a general uplift in mood than to performance changes that are measurable by laboratory tasks. It is worth noting that in a follow up exploratory analysis of only dose days where participants reported not knowing whether they were on the dose or not, this effect on self-rated creativity was not observed (Murphy et al. 2023) This could suggest that either unblinding to condition triggered placebo effects, or that improved subjective effects such as creativity and happiness inform unblinding.

### Placebo and expectancy

Expectancy may play a role in the reports of enhanced creativity by community microdosers (Anderson et al. 2019a; Lea et al. 2019; Prochazkova et al. 2018). In previous reporting of the MDLSD trial results (Murphy et al. 2023), prior to their first dose, participants rated how confident they were that microdosing would alter several different mood and cognitive domains, including creativity. Of these, creativity was the domain that had the highest rated baseline expectancy in both the placebo and LSD groups, indicating this to be a well-known proposed benefit of microdosing. Prospective studies of microdosing have shown that general expectancy among community microdosers is high and that at least some effects may be related to unblinding and expectancy (Kaertner et al. 2021; Polito and Stevenson 2019; Szigeti et al. 2021). Interestingly, one of these studies used a self-blinding protocol and found that while subjective effects appeared to be affected by belief in having taken an active dose, cognitive tasks were not (Szigeti et al. 2021). It also is worth noting that in the present study, where blinding in the placebo group was maintained, only one measure showed a main effect of visit in an enhanced direction – self-rated difficulty of idea generation went down regardless of drug group from the Baseline to the Final visit. None of the more objective tasks showed this pattern. As such it appears that the tasks were not sensitive to expectancy driven placebo effects, but that the problem-solving questionnaire may be.

It is entirely possible that the gap between self-reported impressions of enhanced creativity following psychedelics (in both high and low doses) and the actual creative output during these experiences may be a result of inflated meaning attribution and altered self-concept (Baggott 2015; Girn et al. 2020). If subjective reports of inflated creativity are simply a matter of feeling, then claims that microdosing can enhance one’s productivity and effectiveness at creative pursuits are likely unfounded. However, in terms of feeling creative as a source of eudaimonic pleasure that enhances overall quality of life, these unfounded subjective feelings may still be of benefit to wellbeing and mental health, especially in the context of depressive anhedonia. Future therapeutic research could manipulate set and setting to optimise this effect in conjunction with art therapies to evaluate whether it could be useful in depressive anhedonia.

### Limitations

As mentioned, lack of power may lie behind the absence of creativity effects seen in the laboratory studies of microdosing. The current study was a parallel trial with 40 participants in each treatment group, and previous laboratory studies which tested creativity have included 20 (Bershad et al. 2019), 34 (Cavanna et al. 2022), and 39 (Molla et al. 2023) participants in crossover designs. Subtle microdosing-induced changes may require larger sample sizes to be detectable.

Beyond the limitations stated above there were some procedural limitations to this study. Test-retest reliability of these tasks with this specific combination of items and at three timepoints is also not available and results should be interpreted cautiously with that in mind. Given that the creativity battery was a secondary measure, the battery was not able to be presented at the peak of subjective effects, however acute subjective effects were still evident. Tasks were not counterbalanced within the battery, which could have led to fatigue affecting the later tasks (the CAT and RAT). This study only examined the acute effects of a single dose level (10 µg LSD or below) against placebo, and therefore isn’t indicative of higher dose levels. While some creativity testing has been done at higher microdoses of 20 µg (Bershad et al. 2019; Molla et al. 2023), a functional task such as the CAT has not been done at this dose.

## Conclusions

The present study did not find any evidence that microdosing produced measurable changes to creativity either acutely or two days after the conclusion of a six-week protocol of regular microdosing, across four different assessment modalities. However, the same sample reported feeling more creative on microdosing days. This suggests that if microdosing does have an impact on creativity, it may not be strong enough to produce effects that are measurable with standard tests, or may have no functional output beyond a creative feeling. It may equally be that microdosing has no effect on creativity beyond a general uplift in positive mood, which combined with high expectancy of microdosing’s creative effects generates a feeling of being more creative. Future research should manipulate set and setting to further investigate the nature of this creative feeling and assess whether it can be operationalised for therapeutic benefit.

## Electronic supplementary material

Below is the link to the electronic supplementary material.

Supplementary Material 1

## References

[CR1] Amabile T (1996) Creativity in context. Westview

[CR2] Anderson T, Petranker R, Christopher A, Rosenbaum D, Weissman C, Dinh-Williams L-A, Hapke E (2019a) Psychedelic microdosing benefits and challenges: an empirical codebook. Harm Reduct J 16(1):43. 10.1186/s12954-019-0308-4 31288862 10.1186/s12954-019-0308-4 PMC6617883

[CR3] Anderson T, Petranker R, Rosenbaum D, Weissman C, Dinh-Williams L-A, Hui K, Farb N (2019b) Microdosing psychedelics: personality, mental health, and creativity differences in microdosers. Psychopharmacology 236(2):731–740. 10.1007/s00213-018-5106-2 30604183 10.1007/s00213-018-5106-2

[CR4] Andersson M, Kjellgren A (2019) 20% better with 20 micrograms? A qualitative study of psychedelic microdosing self-rapports and discussions on YouTube. Harm Reduct J 16(1):63. 10.1186/s12954-019-0333-3 31779667 10.1186/s12954-019-0333-3 PMC6883685

[CR5] Baggott MJ (2015) Psychedelics and creativity: A review of the quantitative literature. *PeerJ PrePrints, 3*: e1202v1. 10.7287/peerj.preprints.1202v1

[CR6] Beaty RE, Benedek M, Barry Kaufman S, Silvia PJ (2015) Default and executive network coupling supports creative idea production. Sci Rep 5(1):10964. 10.1038/srep10964 26084037 10.1038/srep10964 PMC4472024

[CR7] Beaty RE, Benedek M, Silvia PJ, Schacter DL (2016) Creative cognition and brain network dynamics. Trends Cogn Sci 20(2):87–95. 10.1016/j.tics.2015.10.004 26553223 10.1016/j.tics.2015.10.004 PMC4724474

[CR8] Bershad AK, Schepers ST, Bremmer MP, Lee R, de Wit H (2019) Acute subjective and behavioral effects of microdoses of lysergic acid diethylamide in healthy human volunteers. Biol Psychiatry 86(10):792–800. 10.1016/j.biopsych.2019.05.019 31331617 10.1016/j.biopsych.2019.05.019 PMC6814527

[CR9] Bowden EM, Jung-Beeman M (2003) Normative data for 144 compound remote associate problems. Behav Res Methods Instruments Computers 35(4):634–639. 10.3758/bf03195543 10.3758/bf03195543 14748508

[CR10] Carhart-Harris RL, Erritzoe D, Williams T, Stone JM, Reed LJ, Colasanti A, Murphy K (2012) Neural correlates of the psychedelic state as determined by fMRI studies with psilocybin. Proc Natl Acad Sci 109(6):2138–2143. 10.1073/pnas.1119598109 22308440 10.1073/pnas.1119598109 PMC3277566

[CR11] Carhart-Harris RL, Muthukumaraswamy S, Roseman L, Kaelen M, Droog W, Murphy K, Orban C (2016) Neural correlates of the LSD experience revealed by multimodal neuroimaging. *Proceedings of the National Academy of Sciences, 113*(17): 4853–4858. 10.1073/pnas.1518377113 10.1073/pnas.1518377113 PMC4855588 27071089

[CR12] Cavanna F, Muller S, de la Fuente LA, Zamberlan F, Palmucci M, Janeckova L, Tagliazucchi E (2022) Microdosing with psilocybin mushrooms: a double-blind placebo-controlled study. Translational Psychiatry 12(1):307. 10.1038/s41398-022-02039-0 35918311 10.1038/s41398-022-02039-0 PMC9346139

[CR13] Christoff K, Irving ZC, Fox KC, Spreng RN, Andrews-Hanna JR (2016) Mind-wandering as spontaneous thought: a dynamic framework. Nat Rev Neurosci 17(11):718–731. 10.1038/nrn.2016.113 27654862 10.1038/nrn.2016.113

[CR14] Daws RE, Timmermann C, Giribaldi B, Sexton JD, Wall MB, Erritzoe D, Carhart-Harris R (2022) Increased global integration in the brain after psilocybin therapy for depression. Nat Med 28(4):844–851 35411074 10.1038/s41591-022-01744-z

[CR15] Diedrich J, Benedek M, Jauk E, Neubauer AC (2015) Are creative ideas novel and useful? Psychol Aesthet Creativity Arts 9(1):35. 10.1037/a0038688

[CR16] Doss MK, Považan M, Rosenberg MD, Sepeda ND, Davis AK, Finan PH, Griffiths RR (2021) Psilocybin therapy increases cognitive and neural flexibility in patients with major depressive disorder. Translational Psychiatry 11(1):574. 10.1038/s41398-021-01706-y 34750350 10.1038/s41398-021-01706-y PMC8575795

[CR17] Ellamil M, Dobson C, Beeman M, Christoff K (2012) Evaluative and generative modes of thought during the creative process. NeuroImage 59(2):1783–1794. 10.1016/j.neuroimage.2011.08.008 21854855 10.1016/j.neuroimage.2011.08.008

[CR18] Fadiman J (2011) The psychedelic explorer’s guide: Safe, therapeutic, and sacred journeys. Park Street 10.1080/02791072.2014.948329 25188706

[CR19] Family N, Vinson D, Vigliocco G, Kaelen M, Bolstridge M, Nutt DJ, Carhart-Harris RL (2016) Semantic activation in LSD: evidence from picture naming. Lang Cognition Neurosci 31(10):1320–1327. 10.1080/23273798.2016.1217030

[CR20] Girn M, Mills C, Roseman L, Carhart-Harris RL, Christoff K (2020) Updating the dynamic framework of thought: Creativity and psychedelics. NeuroImage 213:116726. 10.1016/j.neuroimage.2020.116726 32160951 10.1016/j.neuroimage.2020.116726

[CR21] Guilford JP (1967) The nature of human intelligence. McGraw-Hill

[CR22] Han W, Feng X, Zhang M, Peng K, Zhang D (2019) Mood states and everyday creativity: employing an experience sampling method and a day reconstruction method. Front Psychol 10:1698 31379699 10.3389/fpsyg.2019.01698 PMC6658875

[CR23] Hartogsohn I (2017) Constructing drug effects: a history of set and setting. Drug Sci Policy Law 3:2050324516683325. 10.1177/2050324516683325

[CR24] Hofmann A (1980) LSD, my problem child. McGraw-Hill

[CR25] Holze F, Liechti ME, Hutten NR, Mason NL, Dolder PC, Theunissen EL, Kuypers KP (2020) Pharmacokinetics and pharmacodynamics of lysergic acid diethylamide microdoses in healthy participants. Clin Pharmacol Ther 109(3):658–666. 10.1002/cpt.2057 32975835 10.1002/cpt.2057 PMC7984326

[CR26] Janiger O, de Rios MD (1989) LSD and creativity. J Psychoactive Drugs 21(1):129–134. 10.1080/02791072.1989.10472150 2723891 10.1080/02791072.1989.10472150

[CR27] Kaertner L, Steinborn M, Kettner H, Spriggs M, Roseman L, Buchborn T, Carhart-Harris R (2021) Positive expectations predict improved mental-health outcomes linked to psychedelic microdosing. Sci Rep 11(1):1–11. 10.1038/s41598-021-81446-7 33479342 10.1038/s41598-021-81446-7 PMC7820236

[CR28] Kaufman JC, Plucker JA, Baer J (2008) Essentials of Creativity Assessment. Wiley

[CR29] Kiraga MK, Mason NL, Uthaug MV, van Oorsouw KI, Toennes SW, Ramaekers JG, Kuypers KP (2021) Persisting effects of ayahuasca on empathy, creative thinking, decentering, personality, and well-being. Front Pharmacol 12:721537. 10.3389/fphar.2021.721537 34658861 10.3389/fphar.2021.721537 PMC8517265

[CR30] Kuypers KPC, Riba J, de la Fuente Revenga M, Barker S, Theunissen EL, Ramaekers JG (2016) Ayahuasca enhances creative divergent thinking while decreasing conventional convergent thinking. Psychopharmacology 233(18):3395–3403. 10.1007/s00213-016-4377-8 27435062 10.1007/s00213-016-4377-8 PMC4989012

[CR31] Kuznetsova A, Brockhoff PB, Christensen RH (2017) lmerTest package: tests in linear mixed effects models. J Stat Softw 82:1–26. 10.18637/jss.v082.i13

[CR32] Lea T, Amada N, Jungaberle H (2019) Psychedelic microdosing: a subreddit analysis. J Psychoactive Drugs 52(2):1–12. 10.1080/02791072.2019.1683260 10.1080/02791072.2019.1683260 31648596

[CR33] Lea T, Amada N, Jungaberle H, Schecke H, Klein M (2020) Microdosing psychedelics: motivations, subjective effects and harm reduction. Int J Drug Policy 75:102600. 10.1016/j.drugpo.2019.11.008 31778967 10.1016/j.drugpo.2019.11.008

[CR34] Liu S, Erkkinen MG, Healey ML, Xu Y, Swett KE, Chow HM, Braun AR (2015) Brain activity and connectivity during poetry composition: toward a multidimensional model of the creative process. Hum Brain Mapp 36(9):3351–3372. 10.1002/hbm.22849 26015271 10.1002/hbm.22849 PMC4581594

[CR35] Lubart T, Guignard J-H (2004) The generality-specificity of creativity: a multivariate approach. In: Sternberg RJ, Grigorenko EL, Singer JL (eds) Creativity: from potential to realization. American Psychological Association, pp 43–56. 10.1037/10692-004

[CR37] Mason NL, Mischler E, Uthaug MV, Kuypers KP (2019) Sub-acute effects of psilocybin on empathy, creative thinking, and subjective well-being. J Psychoactive Drugs 51(2):123–134. 10.1080/02791072.2019.1580804 30905276 10.1080/02791072.2019.1580804

[CR36] Mason N, Kuypers K, Reckweg J, Müller F, Tse D, Rios D, Ramaekers B, J (2021) Spontaneous and deliberate creative cognition during and after psilocybin exposure. Translational Psychiatry 11(1):209. 10.1038/s41398-021-01335-5 33833225 10.1038/s41398-021-01335-5 PMC8032715

[CR38] Mednick S (1962) The associative basis of the creative process. Psychol Rev 69(3):220–232. 10.1037/h0048850 14472013 10.1037/h0048850

[CR39] Mednick SA (1968) The Remote associates Test. J Creative Behav 2:213–214. 10.1002/j.2162-6057.1968.tb00104.x

[CR40] Molla H, Lee R, Tare I, de Wit H (2023) Greater subjective effects of a low dose of LSD in participants with depressed mood. Neuropsychopharmacology. 10.1038/s41386-023-01772-4 38042914 10.1038/s41386-023-01772-4 PMC10948752

[CR41] Morse JD, Jeong SH, Murphy RJ, Muthukumaraswamy SD, Sumner RL (2024) Pharmacokinetics and pharmacodynamics of Sublingual Microdosed Lysergic Acid Diethylamide (LSD) in healthy adult volunteers 10.1177/02698811251330747 PMC12371133 40251818

[CR45] Murphy RJ, Sumner RL, Evans W, Menkes D, Lambrecht I, Ponton R, Reynolds L (2021) MDLSD: study protocol for a randomised, double-masked, placebo-controlled trial of repeated microdoses of LSD in healthy volunteers. Trials 22(1):1–15. 10.1186/s13063-021-05243-3 33892777 10.1186/s13063-021-05243-3 PMC8062934

[CR44] Murphy RJ, Sumner R, Evans W, Ponton R, Ram S, Godfrey K, Smith T (2023) Acute mood-elevating properties of microdosed LSD in healthy volunteers: a home-administered randomised controlled trial. Biol Psychiatry 94(6):511–521. 10.1016/j.biopsych.2023.03.013 36997080 10.1016/j.biopsych.2023.03.013

[CR42] Murphy RJ, Godfrey K, Shaw AD, Muthukumaraswamy S, Sumner RL (2024a) Modulation of long-term potentiation following microdoses of LSD captured by thalamo-cortical modelling in a randomised, controlled trial. BMC Neurosci 25(1):7 38317077 10.1186/s12868-024-00844-5 PMC10845757

[CR43] Murphy RJ, Muthukumaraswamy SD, de Wit H (2024b) Microdosing psychedelics: current evidence from controlled studies. Biol Psychiatry: Cogn Neurosci Neuroimaging. 10.1016/j.bpsc.2024.01.002 38280630 10.1016/j.bpsc.2024.01.002

[CR46] National Institutes of Health (2019) *NIH Toolbox Scoring and Interpretation Guide for the iPad*. https://www.nihtoolbox.org/app/uploads/2022/05/Toolbox_Scoring_and_Interpretation_Guide_for_iPad_v1.7-5.25.21.pdf

[CR47] Plucker JA, Beghetto RA (2004) Why creativity is domain general, why it looks domain specific, and why the distinction does not matter. In: Sternberg RJ, Grigorenko EL, Singer JL (eds) Creativity: from potential to realization. American Psychological Association, pp 153–167. 10.1037/10692-009

[CR48] Polito V, Liknaitzky P (2022) The emerging science of microdosing: a systematic review of research on low dose psychedelics (1955–2021) and recommendations for the field. Neurosci Biobehavioral Reviews 139:104706. 10.1016/j.neubiorev.2022.104706 10.1016/j.neubiorev.2022.104706 35609684

[CR49] Polito V, Stevenson RJ (2019) A systematic study of microdosing psychedelics. PLoS ONE 14(2):e0211023. 10.1371/journal.pone.0211023 30726251 10.1371/journal.pone.0211023 PMC6364961

[CR50] Prochazkova L, Hommel B (2020) Altered states of consciousness and creativity. In: Press DD, Cosmelli D, Kaufman JC (eds) Creativity and the wandering mind. Elsevier, pp 121–158. 10.1016/B978-0-12-816400-6.00006-7

[CR51] Prochazkova L, Lippelt DP, Colzato LS, Kuchar M, Sjoerds Z, Hommel B (2018) Exploring the effect of microdosing psychedelics on creativity in an open-label natural setting. Psychopharmacology 235(12):3401–3413. 10.1007/s00213-018-5049-7 30357434 10.1007/s00213-018-5049-7 PMC6267140

[CR52] Raichle ME, MacLeod AM, Snyder AZ, Powers WJ, Gusnard DA, Shulman GL (2001) A default mode of brain function. Proc Natl Acad Sci 98(2):676–682. 10.1073/pnas.98.2.676 11209064 10.1073/pnas.98.2.676 PMC14647

[CR53] Roseman L, Leech R, Feilding A, Nutt DJ, Carhart-Harris RL (2014) The effects of psilocybin and MDMA on between-network resting state functional connectivity in healthy volunteers. Front Hum Neurosci 8:204 24904346 10.3389/fnhum.2014.00204 PMC4034428

[CR54] Runco MA, Jaeger GJ (2012) The standard definition of creativity. Creativity Res J 24(1):92–96. 10.1080/10400419.2012.650092

[CR55] Said-Metwaly S, Van den Noortgate W, Kyndt E (2017) Methodological issues in measuring creativity: a systematic literature review. Creativity Theories–Research-Applications 4(2):276–301. 10.1515/ctra-2017-0014

[CR56] Sayalı C, Barrett FS (2023) The costs and benefits of psychedelics on cognition and mood. Neuron 111(5):614–630. 10.1016/j.neuron.2022.12.031 36681076 10.1016/j.neuron.2022.12.031

[CR57] Seeley WW, Menon V, Schatzberg AF, Keller J, Glover GH, Kenna H, Greicius MD (2007) Dissociable intrinsic connectivity networks for salience processing and executive control. J Neurosci 27(9):2349–2356. 10.1523/JNEUROSCI.5587-06.2007 17329432 10.1523/JNEUROSCI.5587-06.2007 PMC2680293

[CR58] Sessa B (2008) Is it time to revisit the role of psychedelic drugs in enhancing human creativity? J Psychopharmacol 22(8):821–827. 10.1177/026988110809159 18562421 10.1177/0269881108091597

[CR59] Szigeti B, Kartner L, Blemings A, Rosas F, Feilding A, Nutt DJ, Erritzoe D (2021) Self-blinding citizen science to explore psychedelic microdosing. Elife 10:e62878. 10.7554/eLife.62878 33648632 10.7554/eLife.62878 PMC7925122

[CR60] Wakefield JF (1987) The Outlook for Creativity tests. J Creative Behav 25(3):184–193. 10.1002/j.2162-6057.1991.tb01369.x

[CR61] Weintraub S, Dikmen SS, Heaton RK, Tulsky DS, Zelazo PD, Bauer PJ, Wallner-Allen K (2013) Cognition assessment using the NIH Toolbox. Neurology 80(11 Supplement 3):S54–S64. 10.1212/WNL.0b013e3182872ded 23479546 10.1212/WNL.0b013e3182872ded PMC3662346

[CR62] Wießner I, Falchi M, Maia LO, Daldegan-Bueno D, Palhano-Fontes F, Mason NL, Feilding A (2022) LSD and creativity: increased novelty and symbolic thinking, decreased utility and convergent thinking. J Psychopharmacol 36(3):348–359. 10.1177/02698811211069113 35105186 10.1177/02698811211069113

[CR63] Zeng L, Proctor RW, Salvendy G (2011) Can traditional divergent thinking tests be trusted in measuring and predicting real-world creativity? Creativity Res J 23(1):24–37. 10.1080/10400419.2011.545713

[CR64] Zhang M, Wang F, Zhang D (2020) Individual differences in trait creativity moderate the state-level mood-creativity relationship. PLoS ONE 15(8):e0236987 32745087 10.1371/journal.pone.0236987 PMC7398526

