# Supplementary material for: Multimodal creativity assessments following acute and sustained microdosing of lysergic acid diethylamide
Source: Psychopharmacology (Berl). 2024 Sep 5;242(2):337–51. doi: 10.1007/s00213-024-06680-z (PMC11775047; doi:10.1007/s00213-024-06680-z)
Supplement: Supplementary file 1 — Supplementary Material 1 [file 213_2024_6680_MOESM1_ESM.docx]

Supplement to: **Multimodal creativity assessments following acute and sustained microdosing of lysergic acid diethylamide**

**Robin J. Murphy^1*^, Rachael L. Sumner^1^, Kate Godfrey^2^, Acima Mabidikama^3^, Reece P. Roberts^3,4^, Frederick Sundram^5^, and Suresh Muthukumaraswamy^1^**

***Communicating author: Robin J. Murphy – robin.murphy@auckland.ac.nz**

1. **School of Pharmacy, Faculty of Medical and Health Sciences, University of Auckland**
2. **Centre for Psychedelic Research, Division of Psychiatry, Department of Brain Sciences, Imperial College London**
3. **School of Psychology, Faculty of Science, University of Auckland**
4. **Centre for Brain Research, University of Auckland**
5. **Department of Psychological Medicine, Faculty of Medical and Health Sciences, University of Auckland, New Zealand**

# ­­­­­

# Methods

## Inclusion and exclusion criteria

**Table S1: Full inclusion criteria**

|  | **Inclusion criteria** |
| --- | --- |
| Consent | Willing and able to give informed consent for participation in the trial, reconfirmed verbally at each study visit. |
| Demographics |  |
| Age | 25-60 years |
| Sex | Male |

**Table S2: Full exclusion criteria**

|  | **Exclusion criteria** |
| --- | --- |
| Consent/communication | Inability to speak or read English |
| Physiological health: |  |
| Diagnosis | Unstable medical or neurologic condition as assessed by study physician |
| Lab work | Significant renal or hepatic impairment |
| Vital signs | Cardiovascular conditions including abnormal heart rate seen by ECG  Resting blood pressure not exceeding 160 mmHg systolic and 90 mmHg diastolic  Body weight between 50-120 kg |
| Mental health: |  |
| Diagnosis | Lifetime history of major depressive disorder, schizophrenia, or other psychotic disorders, or bipolar I or II disorder as assessed by the Mini International Neuropsychiatric Interview (MINI)  Current diagnosis of PTSD, anxiety and panic disorders, OCD, dysthymic disorder, anorexia, and bulimia as assessed by the MINI |
| Current risk | Elevated of suicide as determined by study psychiatrist using the Columbia-Suicide Severity Rating Scale (C-SSRS)  Elevated risk of developing psychosis as determined by study psychiatrist using the Comprehensive Assessment of At Risk Mental States (CAARMS) |
| Family diagnosis | First degree relatives diagnosed with schizophrenia or other primary psychotic disorder, or bipolar I or II disorder |
| Medication | Current use of any prescribed psychotropic medication |
| Substance use | Substance use disorder in the previous 3 months as assessed with a New Zealand modified version of the NIDA-Modified ASSIST (NM-ASSIST)  Failed breathalyser and/or multipanel drug urine tests at screening with one follow up in trial  Use of serotonergic psychedelic drugs in the last year  Lifetime history of psychedelic microdosing |

## Alternate Uses Test

Instructions were adapted from Gilhooley *et al.* (2007):

You will be asked to produce as many different uses as you can think of, which are different from the normal use, for a number of common objects. For example, the common use for a newspaper is for reading, but it could also be used for swatting flies, to line drawers, to make a paper hat and so on. You are to try to produce possible uses which are different from the normal one and different in kind from each other. You will have two minutes for each item. Press the arrow button when you are ready to start.

The three versions of the AUT task were:

1. Chair, brick, towel
2. Ball, knife, glove
3. Cup, tyre, pencil

## Remote Associates Task

Instructions were adapted from a previously used test battery [1], given in Figure 1.


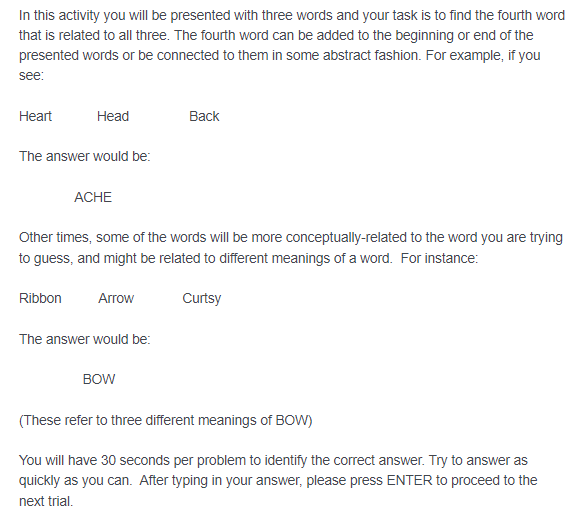


**Figure S1: Instructions for the RAT adapted from Mueller (2012)**

# Results

## Summary statistics

Table S3: Mean and interquartile range for each measure

|  | **Placebo** | | |  | **LSD** | | |
| --- | --- | --- | --- | --- | --- | --- | --- |
| Measure | **Baseline, mean (IQR)** | **Treatment, mean (IQR)** | **Final, mean (IQR)** |  | **Baseline, mean (IQR)** | **Treatment, mean (IQR)** | **Final, mean (IQR)** |
| AUT Elaboration | 5.6 (3.3,7) | 5.3 (3.7,6.3) | 5.3 (2.9,7.2) |  | 5 (3.6,5.8) | 4.9 (3,6) | 5.4 (2.9,6.7) |
| AUT Flexibility | 5.8 (4.3,6.8) | 5.7 (4.3,6.7) | 5.6 (4.3,7) |  | 5.4 (4,6.7) | 5.1 (4,6.5) | 5.2 (4,6.3) |
| AUT Fluency | 7.2 (5.3,8.8) | 6.8 (5.7,8) | 7.2 (5,9.1) |  | 6.8 (5,8.7) | 7.1 (5.2,8.8) | 6.8 (4.7,8.6) |
| AUT Originality | 4.6 (2.5,6.3) | 5.2 (3.3,7) | 4.8 (3.2,6.8) |  | 4.4 (2,6) | 4.7 (3,6.3) | 4.1 (2.9,5.4) |
| RAT Correct | 8 (6,10) | 8.1 (6.8,10) | 8.2 (7,10) |  | 7.8 (6,10) | 7.8 (6,9) | 7.8 (6,9) |
| RAT Attempted | 14.7 (12.8,17.2) | 15.1 (12.8,18) | 15.2 (13,18) |  | 14.2 (11,17) | 15.1 (11.8,18) | 14.2 (12.5,17) |
| CAT Creativity | 5.4 (4.5,6.2) | 6.1 (5.5,6.9) | 5.7 (5.2,6.5) |  | 5.5 (4.8,6.4) | 5.5 (4.8,6.3) | 5.7 (4.8,6.7) |
| CAT Technical Goodness | 4.7 (3.9,5.3) | 5.3 (4.4,6.2) | 5.2 (4.5,6.3) |  | 4.9 (4.1,5.4) | 5 (4.3,5.9) | 4.8 (3.7,5.8) |
| EPSQ idea generation | 0.7 (-19.5,18.2) | - | -12.8 (-20.2,-8.8) |  | 2.6 (-12,17) | - | -17.9 (-30,-8.5) |
| EPSQ idea effectiveness | 13.1 (0,27.2) | - | 21 (10,27.2) |  | 19.1 (12.8,27.2) | - | 18.8 (9.8,34) |
| *Note:* AUT – Alternate Uses Test; CAT – Consensual Assessment Technique; EPSQ – Everyday Problem-Solving Questionnaire; RAT – Remote Associates Task; IQR – Interquartile range. | | | | | | | |

# References

1. Mueller, S., *The PEBL manual: Programming and usage guide for the Psychology Experiment Building Language PEBL, Version 0.13*. 2012, Lulu Press: Raleigh, NC.
